# Modification of preoperative radiochemotherapy for esophageal cancer (CROSS protocol) is safe and efficient with no impact on surgical morbidity

**DOI:** 10.1007/s00066-020-01594-z

**Published:** 2020-02-13

**Authors:** Matthias Paireder, Gerd Jomrich, Ivan Kristo, Reza Asari, Erwin Rieder, Andrea Beer, Aysegül Ilhan-Mutlu, Matthias Preusser, Rainer Schmid, Sebastian F. Schoppmann

**Affiliations:** 1 Department of Surgery, Upper GI Service, Comprehensive Cancer Center GET-Unit, Medical University of Vienna, Spitalgasse 23, 1090 Vienna, Austria; 2 Department of Pathology, Comprehensive Cancer Center GET-Unit, Medical University of Vienna, Vienna, Austria; 3 Clinical Division of Oncology, Department of Medicine I and Comprehensive Cancer Center, GET-Unit, Medical University of Vienna, Vienna, Austria; 4 Department of Radiation Oncology, Comprehensive Cancer Center GET-Unit, Medical University of Vienna, Vienna, Austria

**Keywords:** Neoadjuvant radiochemotherapy, Esophagus, Esophageal resection, CROSS protocol, Prognosis

## Abstract

**Purpose:**

Neoadjuvant radiochemotherapy (RCTH) is proven to be highly effective in the treatment of esophageal cancer (EC). We investigated oncological outcome and morbidity in patients treated with a modified CROSS protocol followed by esophagectomy at our institution.

**Methods:**

Patients with EC receiving neoadjuvant RCTH with paclitaxel and carboplatin and concurrent radiotherapy (46 Gy) followed by esophagectomy were included in this retrospective analysis. Histopathological response, overall survival (OS) and recurrence-free interval (RFI) as well as perioperative morbidity were investigated.

**Results:**

Thirty-six patients (86.1% male, mean age 61.3 years, standard deviation 11.52) received neoadjuvant RCTH before surgery. Sixteen patients (44.4%) were treated for squamous cell cancer, whereas 20 patients (55.6%) had adenocarcinoma. The majority (75%) underwent abdominothoracic esophageal resection. Major complications occurred in 7 patients (19.5%) including anastomotic leakage in 4 patients (11.1%). A R0 resection was achieved in 97.2%. A complete pathological remission was seen in 13 patients (36.1%). Major response, classified as Mandard tumor regression grade 1 and 2, was found in 26 patients (72.2%). Median OS and RFI were not reached.

**Conclusions:**

Neoadjuvant radiotherapy with 46 Gy and concomitant chemotherapy with paclitaxel and carboplatin for the treatment of locally advanced esophageal carcinoma is safe and effective. The results of this modified radiotherapy protocol are encouraging and should be considered in future patient treatment and study designs.

## Background

Esophageal cancer (EC) is a rare tumor entity associated with a dramatically growing incidence [1]. Despite improvement in therapy, patients are still confronted with poor prognosis [2]. In locally advanced stage, the multimodal approach gained significant relevance in the treatment of EC [3, 4].

Importantly, the randomized controlled CROSS trial emphasized the advantage of a neoadjuvant radiochemotherapy (RCTH) regimen with paclitaxel and carboplatin and concurrent radiotherapy with 41.4 Gray (Gy) over surgery alone [5]. Furthermore, the authors highlighted that patients with presence of adenocarcinoma (AC) as well as squamous cell carcinoma (SCC) benefited from neoadjuvant chemoradiation. However, a higher pathological complete response (pCR) rate was obtained in patients presenting with SCC [5]. Confirming these results, Toxopeus et al. investigated outcomes of patients treated within and outside the CROSS randomized controlled trial [6]. Based on their findings, the authors encouraged extrapolation of the CROSS treatment into daily practice [6].

As a consequence, the CROSS scheme has also been established at our tertiary academic referral center enriching our perioperative treatment concepts. Five years after the implementation of this neoadjuvant protocol at the Medical University of Vienna, this retrospective study was performed to evaluate oncological results such as pathological response rates and survival data as well as the impact of RCTH on perioperative outcomes.

## Methods

### Patients

All patients, who underwent neoadjuvant radiochemotherapy and/or esophageal resection for esophageal cancer after neoadjuvant treatment according to the modified CROSS protocol at the Department of Surgery, Medical University of Vienna, between the years 2013 and 2018, were included in this analysis.

Clinical data were obtained from an institutional prospective database. Patients were followed-up on a 3-monthly basis for the first 2 years and every 6 months until year 5 after surgery. In order to optimize data accuracy and reduce the number of patients lost to follow-up, patients were contacted to evaluate the current status if information was missing. The ethic commission of the Medical University of Vienna approved the study (EK2248/2017) and the study was conducted in accordance with the Declaration of Helsinki Principals. Individual informed consent was not acquired, due to study design and national regulations.

### Staging

Each patient underwent a multidisciplinary tumor board decision confirming preoperative treatment. Staging included computed tomography scan (all patients) and fluorodeoxyglucose positron emission tomography–computed tomography (FDG PET-CT) in selected cases (*n* = 15) as well as positron emission tomography–magnetic resonance imaging (FDG PET-MRI; *n* = 1) [7]. Tumor staging was performed according to the tumor, node, metastasis (TNM) classification of the 7th Edition of the AJCC Cancer Staging Manual [8]. Histopathological response was assessed according to the tumor regression grading (TRG) system according to Mandard et al. [9]. Concerning AC location was classified following the Siewert classification of the adenocarcinoma of the esophagogastric junction (AEG) [10].

### Eligibility criteria

**Fig. 1:**
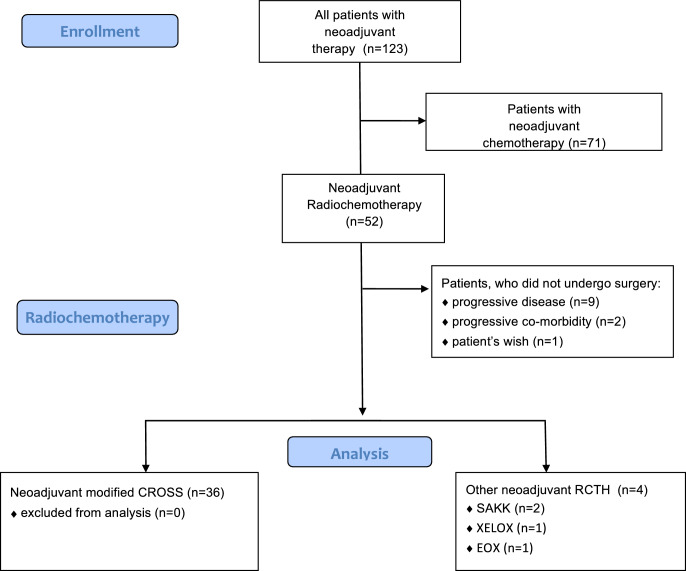
Flow chart depicting the enrollment process. Modified *CROSS* neoadjuvant chemoradiotherapy plus surgery versus surgery alone for esophageal or junctional cancer (CROSS trial), *SAKK* Swiss Group for Clinical Cancer Research phase II trial (SAKK 75/08), *XELOX* capecitabine and oxaliplatin, *EOX* epirubicin, oxaliplatin and capecitabine

All patients who underwent neoadjuvant RCTH followed by esophageal resection for esophageal carcinoma (AC as well as SCC) were included in this analysis. All cases presented at the multidisciplinary tumor board during the study period (January 2013 to November 2018) were screened for eligibility. Patients who did not receive surgery due to progressive disease, increase of comorbidities or other reasons were not included. Also patients who did not receive any radiotherapy at all were not included. The enrollment process is depicted in Fig. 1.

### Radiochemotherapy

Most of the patients (34/36) received neoadjuvant radiochemotherapy at our center. RCTH was applied according to international guidelines on treatment of esophageal cancer [11, 12].

Deep inspiration breathhold technique was used for planning-computed tomography and during radiotherapy whenever the patient tolerated it.

Three dimensional (3D) treatment planning was based on the contouring of target volumes and organs at risk. Imaging used was a computed tomography scan in treatment position. Clinical target volume (CTV) was defined by the primary tumor and enlarged lymph nodes. Additional margin was approximately 3 cm longitudinal and 0.5–1 cm radial. The treatment decision was based on all available diagnostic information: endoscopy, contrast enhanced computed tomography, FDG-PET scan [7]. Planning target volume (PTV) was generated by adding a margin of 1 cm to CTV to compensate for setup errors. The following organs at risk were contoured: lungs, heart, spinal cord, kidneys, liver, stomach and peritoneal cavity (representative for small and large bowel). A multiple field technique combined with multileaf collimation was used to obtain optimal target volume coverage and minimum dose to normal tissue. Thirty-four (94.4%) patients had a 3D conformal treatment planning, 1 patient received a volumetric modulated arc therapy (VMAT) and in 1 patient the treatment technique is unknown. A total dose of 46 Gy, specified at the International Commission on Radiation Units (ICRU) 50/62 reference point, was given in fractions of 2 Gy, 5 days a week with a linear accelerator (beam energy ≥10 MV). Radiotherapy (RT) was combined with a radiosensitizing medication (paclitaxel 50 mg/m^2^ body surface and carboplatin 2 mg × ml ^−1^ × min^−1^ area under the curve up to a total of 5 cycles at weekly intervals).

### Surgery

All esophageal resections were performed at our tertiary center. According to tumor location patients underwent either abdominothoracic esophageal resection (Ivor Lewis esophagectomy), including hybrid minimal invasive esophagectomy, thoracoabdominocervial resection (McKeown esophagectomy) or transhiatal extended gastrectomy [13–15]. Morbidity was classified according to the Clavien/Dindo (C/D) classification [16].

### Statistics

Age is described as mean and standard deviation (SD). Other continuous variables are described as medians and quartiles due to nonnormal distributions. Interquartile range (IQR) was stated, when applicable. Body mass index (BMI) was calculated with dividing weight in kilograms by height in meters squared. Categorical variables are described as counts and percentages. Overall survival and relapse-free interval was estimated using the Kaplan–Meier method. Furthermore, the 95% confidence interval (95% CI) is reported, if computable. Data were statistically analyzed using GraphPad Prism 7.0 (GraphPad, Inc., La Jolla, CA, USA).

## Results

### Demographics

Fifty-two patients underwent neoadjuvant RCTH. After excluding patients according to eligibility criteria (including 9 [17.3%] subjects not undergoing surgery due to progression of disease) 36 patients remained for final analysis (Fig. 1). Mean age was 61.3 (SD 11.52), 31 (86.1%) patients were male and the median BMI was 24.2 (IQR 21.3–26.8) kg/m^2^.

Sixteen patients (44.4%) were treated for SCC, whereas 20 (55.6%) patients presented an AC (14 [38.9%] AEG I, 6 [16.7%] AEG II by Siewert classification). All patients were at least staged cT3 or nodal positive. Additional demographic details are depicted in Table 1.

**Table 1:** Demographics and tumor-related details

Variable	Modified CROSS (*n* = 36)
*Age, years°*	61.3 (11.52)
*Body mass index*^a^	24.7 (21.5–26.4)
*Gender*	
Male	31 (86.1)
Female	5 (13.9)
*ASA classification*	
ASA 1	4 (11.1)
ASA 2	19 (52.8)
ASA 3	13 (36.1)
*Tumor location*	
Thoracic	16 (44.4)
Siewert type I	14 (38.9)
Siewert type II	6 (16.7)
*Tumor histology, No. (%)*	
Adenocarcinoma	20 (55.6)
Squamous cell carcinoma	16 (44.4)
*Clinical tumor staging*	
cT2	5 (13.9)
cT3	29 (80.6)
cT4a	2 (5.6)
Clinical nodal staging	
cN0	2 (5.5)
cN1	34 (94.4)
cN2	0

*CROSS* neoadjuvant radiochemotherapy; *ASA* American Society of Anesthesiologists

Values in parentheses are percentages unless indicated otherwise; °values are mean (standard deviation)

^a^values are median (interquartile range)

All patients received neoadjuvant RCTH according to the modified CROSS protocol. A dose of 46 Gy was applied in 32 patients. One patient was treated with 34.2 Gy due to toxicity of chemotherapy, 1 patient had 41.4 Gy, 1 patient had 50 Gy, 1 patient had 46 Gy with external beam therapy plus additional high dose rate (HDR) brachytherapy to the residual tumor. All 5 cycles of concomitant chemotherapy were administered in 19 (52.8%) patients. Eleven patients (30.6%), 4 (11.1%) and 2 (5.6%) patients received only 4 cycles, 3 cycles and 2 cycles of CTH, respectively. The reduction of chemotherapy cycles was caused by leukopenia in 12 patients (33.3%), by thrombopenia in 2 patients (5.6%), in 1 patient (2.8%) due to infection and in 2 patients (5.6%) the reduction of cycles was caused by delay (without treatment toxicity). Information about chemotoxicity is displayed in Table 2. There were no radiotherapy-associated side effects (acute or chronic) exceeding adverse events grade II.

**Table 2:** Adverse events during radiochemotherapy

	All (*n* = 36)
*Events of grade 3 events according to CTCAE*	
Leukopenia	12 (33.5%)
Thrombopenia	2 (5.6%)
Infection	1 (2.8%)
*Other reason for reduced CTH*	
Delay without toxicity	2 (5.6%)

*CTCAE* Common Terminology Criteria for Adverse Events

Values in parentheses are percentages

### Perioperative details and morbidity

Patients without tumor progression proceeded to surgery within a median of 7 weeks (range 2–27 weeks) after completion of radiochemotherapy. The majority (27 [75%] patients) underwent abdominothoracic esophageal resection (Ivor Lewis esophagectomy), including 1 hybrid minimal invasive esophagectomy. Thoracoabdominocervial resection (McKeown esophagectomy) was done in 3 (8.3%) patients due to tumor location above the carina. Six patients (16.7%) underwent transhiatal extended gastrectomy due to an AEG II position of the tumor.

Major complications (Clavien/Dindo IIIa, b and IVa, b) occurred in 7 patients (19.5%). Anastomotic leakage and pulmonary complications were seen in 4 patients (11.1%) and 1 patient (2.8%), respectively. Median operation time was 292.5 min (IQR 255–350 min). Reoperation was indicated in 4 patients due to anastomotic leakage. Endoscopic treatment (balloon dilatation of the pylorus) was performed in another patient due to pyloric spasm. For more perioperative details see Table 3.

**Table 3:** Perioperative details and morbidity

Variable	All (*n* = 36)
*Clavien/Dindo grade*	
I and II	3 (8.4)
IIIa, b and IVa, b	7 (19.5)
V	0 (–)
*Anastomotic leakage*	4 (11.1)
*Gastric conduit necrosis*	0 (–)
*Reoperation*	4 (11.1)
*Endoscopic intervention*	1 (2.8)
*Pulmonary complication*	1 (2.8)
*Operation duration*^a^	292.5 (255.0–350.0)
*ICU days*^a^	3 (2.0–5.0)
*Length of hospital stay*^a^	13 (10–15.8)

Values in parentheses are percentages unless indicated otherwise

Operation duration is displayed in minutes

*ICU* (Intensive Care Unit) stay and Length of hospital stay in days

^a^ Values are median (interquartile range)

### Histopathological results and survival

A R0 resection was achieved in 35 patients (97.2%). One patient (2.8%) showed a microscopically involved resection margin. Median number of lymph node removal was 19 (range 7–43). A complete pathological remission (ypT0, N0) occurred in 13 patients (36.1%). TRG 1 (ypT0) and 2 (presence of rare cancer cells scattered through the fibrosis), classified as major response was found in 26 patients (72.2%). TRG 5 (absence of regressive changes) or even progression regarding the preoperative T stage was not seen in this study population. For more histopathological details see Table 4.

**Table 4:** Histopathological results

Variable	Modified CROSS (*n* = 36)
*Pathologic tumor stage*	
ypT0	13 (36.1)
ypT1	2 (5.6)
ypT2	6 (16.7)
ypT3	15 (41.7)
ypT4a	0
*Pathologic nodal stage*	
ypN0	22 (61.1)
ypN1	9 (25)
ypN2	3 (8.4)
ypN3	2 (5.6)
*Tumor grading*	
Well differentiated (G1)	0
Moderately differentiated (G2)	13 (36.1)
Poorly differentiated (G3)	10 (27.8)
*Surgical margin status*	
Clear	35 (97.2)
Microscopically involved (R1)	1 (2.8)
Macroscopically involved (R2)	0
Tumor regression grade (Mandard)	
TRG 1	13 (36.1)
TRG 2	13 (36.1)
TRG 3	7 (19.4)
TRG 4	3 (8.3)

Values in parentheses are percentages; modified CROSS, neoadjuvant radiochemotherapy

*TRG* Mandard tumor regression grade

Median follow-up was 13 months (IQR 8.6–19.3 months). In this follow-up period median overall survival (OS) and median recurrence-free interval (RFI) was not reached. The causes of death were tumor progression in 3 patients. Nontumor related causes of death were reduced general condition, a different malignancy and late infection due to anastomotic fistula after 2 years.

**Fig. 2:**
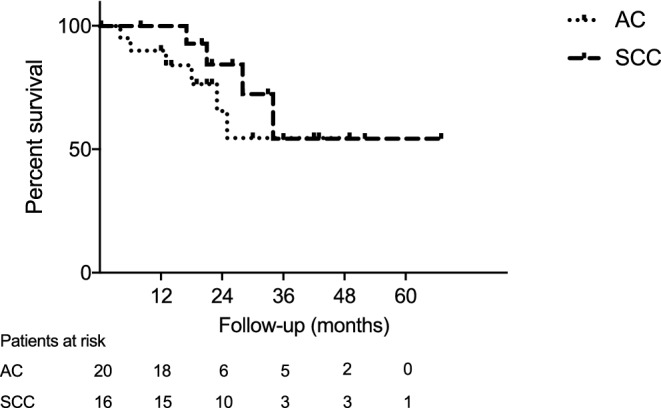
Kaplan–Meier analysis of overall survival after neoadjuvant radiochemotherapy followed by surgery. *AC* adenocarcinoma, *SCC* squamous cell carcinoma

**Fig. 3:**
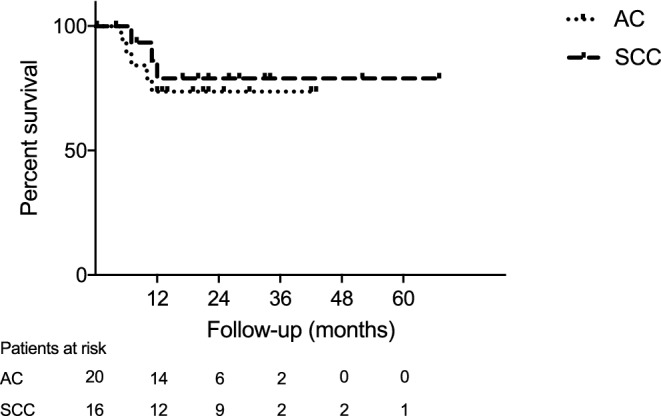
Kaplan–Meier analysis of recurrence-free interval after neoadjuvant radiochemotherapy followed by surgery.* AC* adenocarcinoma, *SCC* squamous cell carcinoma

Tumor progression was found in 7 patients (19.4%). Two patients (5.6%) were diagnosed with malignant pleural effusion, whereas 5 patients (13.9%) developed distant metastasis. No patient had locoregional recurrence. Comparing the histological groups (AC and SCC), there were no significant differences regarding OS and RFI. The Kaplan–Meier curves of OS and RFI are depicted in Figs. 2 and 3.

## Discussion

This is a single-center experience reporting the implementation of the modified CROSS protocol into the neoadjuvant treatment setting of locally advanced EC. This study shows that introduction of a modified study protocol into daily practice is safe and feasible.

Comparing our findings with earlier studies, the presented results are in line with the current literature [5, 17].

Dosage of RT differs throughout literature in neoadjuvant settings [18, 19]. This does not only result in difficulties comparing studies precisely, it may also impact clinical results. Adenis and Mariette postulated that the moderate dose of the CROSS trial (41.4 Gy) may have improved the safety of the subsequent surgery [20]. The dosage used in this study (46 Gy) was comparable with the dose used in the NEOSCOPE phase II trial (45 Gy) [18]. Interestingly, contrary to other reports, we did not observe increased morbidity using a higher dosage of radiation [19]. This may be explained by a considerable difference in dosage of radiation (50.4 Gy vs. 46 Gy in this publication) [19].

Median time to surgery in this study was 7 weeks, although one patient received delayed surgery due to personal reasons. This correlates well to the literature, where surgery within around 8 weeks is suggested. Recently, a study reported no impact of varying interval (time to surgery <8 weeks compared to >8 weeks) on oncological outcome or postoperative morbidity after RCTH [21]. Subsequently, a meta-analysis did not show any benefit of a prolonged interval (mainly cutoff of 7–8 weeks) between RCTH and surgery [22]. Moreover, this systematic review concluded that a prolonged period might increase the risk for anastomotic complications.

Regarding the CR rate, the results in this study are comparable to earlier studies [5, 17]. The high 36.1% CR rate found in this study supports the idea that those patients may benefit from a meticulous surveillance protocol after neoadjuvant RCTH. The Dutch-based SANO study group published a sophisticated study protocol, which might prove surveillance as a possible alternative to esophagectomy in this patient group [23].

Beyond dosage other parameters of radiotherapy may impact oncological and perioperative outcome. Oppedijk et al. analyzed patterns of local recurrence in the CROSS trials. Radiotherapy led to a reduction of locoregional recurrences from 34% (surgery) to 14% (radiotherapy + surgery), but still half of these recurrences (8%) were found at the edge or outside the radiation volume. In the present cohort no locoregional recurrence was found. The short median follow-up of 13 months compared to 45 months of the CROSS cohort may be an explanation. However, lowering these recurrences by increasing radiation volumes has to be put against the risk of worsening perioperative morbidity [24].

Radiotherapy technique itself might influence the risk of local recurrence and long-term morbidity. In comparison of the new technique of intensity-modulated radiation therapy (IMRT) versus standard 3D conformal radiotherapy Lin et al. found on a large cohort (*n* = 676) better overall survival, better locoregional control, fewer noncancer-related and cardiac deaths for IMRT [25].

In our study treatment was mainly 3D conformal radiotherapy. Nevertheless beam arrangement was adjusted to keep dose exposure to the heart as low as possible. Second, deep inspiration breathhold technique led to an improved quotient of lung and planning target volume (lung/PTV) and thus minimized lung volume exposed to 20 Gy.

Furthermore, upcoming radiotherapy machines delivering proton beams enable treatment directly to the target region without collateral damage of adjacent organs, e.g., lungs and heart. Makishima et al. compared morbidity rates in the heart and lungs in a proton group versus an X‑ray group and found lower morbidity rates in the proton group, consistent with lower doses to heart and lungs in the proton group [26]. Proton treatment would offer reduced dose to organs at risk adjacent to the planning target volume, but unfortunately proton therapy was not available for esophageal treatment during study period. However in this study, the beam arrangement was optimized to spare dose to the heart and deep inspiration breathhold technique was used to reduce dose to the lungs.

It is important to stress several limitations associated with a retrospective single center experience. First, there is only a small sample size included in this analysis. Nevertheless, the perioperative and surgical technique as well as the radiotherapy technique at the medical university of Vienna did not change during the study period and reflects a stable treatment algorithm.

Still, a subgroup analysis is not reasonable for this cohort. Second, this study only included patients who proceeded to surgery. For a comprehensive analysis of neoadjuvant radiotherapy, all patients receiving RCTH should be taken into account. However, including patients in a consecutive manner limited a potential selection bias.

However, the aim of the study was to evaluate the impact of RCTH on surgery and its postoperative course. We were able to demonstrate that the extrapolation with a minor adaption of a new treatment protocol can be safely done in a tertiary setting.

Still, there are questions which need to be answered: first, it is not known if RCTH plays a permanent part in the neoadjuvant therapy of AC of the esophagus [27]. Maybe a CTH regime combined with PD1/PDL1 checkpoint inhibitors or other novel agents may enhance efficacy and improve outcome (ICONIC trial, NCT03399071). Second, dosage in RCTH somewhat differs throughout the literature. Furthermore, there are several other aspects (e.g., definition of gross tumor volume and planning target volume) which influence RT and its impact on oncological and perioperative outcome. And third, it is still unknown what we should offer patients with clinical complete response after RCTH. The results of the SANO study are expected with great interest [23].

## Conclusions

Neoadjuvant radiotherapy with 46 Gy and concomitant chemotherapy with paclitaxel and carboplatin for the treatment of locally advanced esophageal carcinoma shows favorable pathological complete response rates without negative impact of surgical morbidity. Our data support the idea of the high significance of radiotherapy for local tumor treatment combined with chemotherapy for a completing systemic effect. The results of this modified radiotherapy protocol are encouraging and should be considered in future patient treatment and study designs.
